# Cellular Core/Sheath Filaments with Thermoresponsive Vacuum Cavities for Prolonged Passive Temperature‐Adaptive Thermoregulation

**DOI:** 10.1002/advs.202412448

**Published:** 2025-01-07

**Authors:** Jiayi Sui, Shoukun Jiang, Jinhao Peng, Zhanxiao Kang, Jintu Fan

**Affiliations:** ^1^ School of Fashion and Textiles The Hong Kong Polytechnic University Hung Hom Kowloon Hong Kong 999077 China; ^2^ Research Centre of Textiles for Future Fashion The Hong Kong Polytechnic University Hung Hom Kowloon Hong Kong 999077 China; ^3^ College of Chemistry and Environmental Engineering Shenzhen University Shenzhen 518055 P. R. China; ^4^ Future Intelligent Wear Centre The Hong Kong Polytechnic University Hung Hom Kowloon Hong Kong 999077 China; ^5^ Research Institute of Sports Science and Technology The Hong Kong Polytechnic University Hung Hom Kowloon Hong Kong 999077 China

**Keywords:** droplet‐based microfluidics, passive thermoregulation filament, phase change materials, temperature‐adaptive thermal conductivity, vacuum cavity

## Abstract

Acting as the interface between the human body and its environment, clothing is indispensable in human thermoregulation and even survival under extreme environmental conditions. Development of clothing textiles with prolonged passive temperature‐adaptive thermoregulation without external energy consumption is much needed for protection from thermal stress and energy saving, but very challenging. Here, a temperature‐adaptive thermoregulation filament (TATF) consisting of thermoresponsive vacuum cavities formed by the temperature‐responsive volume change of the material confined in the cellular cores of the filament is proposed. Using a droplet‐based microfluidic system, the cellular core/sheath filament using octadecane (OD) as a temperature‐responsive volume‐changing material to form droplet cellular cores within the thermoplastic polyurethane (TPU) sheath is fabricated. It is found that the fabric made of TATF has a remarkable temperature adaptive thermal conductivity, which increases by 83% as the mean fabric temperature increases from 20 °C to 35 °C, due to the volume change of vacuum cavities in the cellular cores of the filament in response to temperature. TATF fabrics have no problem associated with undesirable appearance changes or leakage of encapsulated molten materials as some existing thermoregulatory textiles do, and can therefore have wide applications in functional clothing for prolonged passive personal thermal management.

## Graphical abstract

A cellular core/sheath filament is fabricated by droplet‐based microfluidics, which has temperature‐adaptive thermal conductivity due to the thermoresponsive vacuum cavities formed within the cellular cores. The fabrics produced by the proposed filament can preserve body heat in cold environments and facilitate body heat dissipation in hot environments, offering an effective and prolonged strategy for passive personal thermal management.

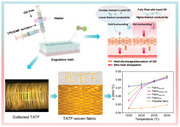

## 1 Introduction

With the increasing global warming, extreme climates, such as heat waves and cold waves, happen more frequently,^[^ ^1^ ^]^ leading to increased energy costs for HVAC (heating, ventilation, and air conditioning) and/or serious temperature‐related illnesses (e.g., heat rash and frostbite) that threaten human health.^[^ ^2^ ^]^ Thus, substantial effort has been directed to develop thermoregulatory textiles for building energy saving and protection from heat/cold stress, through various means including air convection,^[^ ^3^, ^4^ ^]^ liquid convection,^[^ ^5^ ^]^ asymmetric infrared radiation,^[^ ^6^, ^7^ ^]^ sweat/water evaporation^[^ ^8^, ^9^ ^]^ and phase change materials (PCMs).^[^ ^10^, ^11^, ^12^ ^]^ Cooling garments based on blowing air or pumping cold water into the clothing microclimate consume external power, add garment weight, and/or generate much noise; radiation cooling garments lose their effectiveness in extremely hot environments; evaporative cooling garments are not effective in high‐humidity environments; the thermal regulation effect of conventional PCM garments based on the absorption and release of latent heat is only transient with very short working time.

Textiles with prolonged passive temperature‐adaptive thermoregulation are attractive because they consume no external energy consumption and provide lasting thermal regulation effects. To achieve this, shape memory alloys (SMA) were incorporated into clothing, which expanded or shrank to tune the air gaps within clothing so as to change the thermal insulation in response to the changes of environmental temperature.^[^ ^13^, ^14^ ^]^ However, SMA‐based thermoregulatory clothing was not very effective as SMA is heat conductive and heavy. Shape memory polymer was coated onto textile fabrics to modulate the size of fabric pores for thermal regulation, however durability was a major issue as the initial shape of such fabric could not be restored after limited usage.^[^ ^15^ ^]^ Moreover, Janus fabric made of yarns having different thermal expansion on the two sides was developed to generate tunable air gaps within the fabric for variable thermal insulation,^[^ ^16^ ^]^ however clothing made of such fabrics is bulky and only suitable for use in cold environments. The strategy of asymmetric thermal expansion of Janus materials was also applied to fabricate metal coated film actuators on clothing to create multimodal (viz. convection, radiation, and sweat evaporation) thermoregulation, yet the practicality of such clothing is hampered by its high cost and inconvenience in wear.^[^ ^7^ ^]^ Besides, a soft robotic textile was developed to adjust the clothing air gap through the volume change caused by the reversible phase transition of a low boiling point fluid, but it was only suitable for fire protective clothing due to the relatively high boiling temperature (61 °C) and the bulky clothing construction after inflation.^[^ ^17^ ^]^

Ideal temperature‐adaptive thermoregulation textiles should possess prolonged thermoregulation responding to varying environmental conditions without compromising appearance and wearing comfort. Here, we produced a temperature‐adaptive thermoregulation filament (TATF) consisting of thermoresponsive vacuum cavities formed by the temperature‐responsive volume change of the material confined in the cellular cores of the filament. In cold environments, the shrinkage of the confined material generates vacuum cavities in the filament, reducing its thermal conductivity and thereby increasing thermal insulation. In hot environments, the thermal expansion of the confined material fills up the vacuum cavities, resulting in high thermal conductivity and reduced thermal insulation for heat dissipation. Octadecane (OD) was chosen as a temperature‐responsive volume‐changing material because it has very different densities in its liquid (*ρ* = 0.7770 g mL−1) and solid forms (*ρ =* 0.9300 g mL−1).^[^ ^18^ ^]^ In addition, OD is non‐toxic and biocompatible, and does not cause allergic reactions. Consequently, OD is extensively used in the cosmetic, medical, and food industries.^[^ ^19^, ^20^, ^21^ ^]^ Furthermore, droplet‐based microfluidics was applied to produce the TATF with cellular OD cores and TPU sheath. Compared with conventional core–sheath PCM filaments, the novel cellular TATF filament not only prevents the leakage of molten OD, but also have long‐lasting thermoregulation function owing to its unique ability of modulating thermal conductivity with its temperature as a result of temperature responsive volume change of the vacuum cavities within the OD cells of the filament.

## 2 Experimental Section

### 2.1 Fabrication and Optimization of TATF

**Figure 1:**
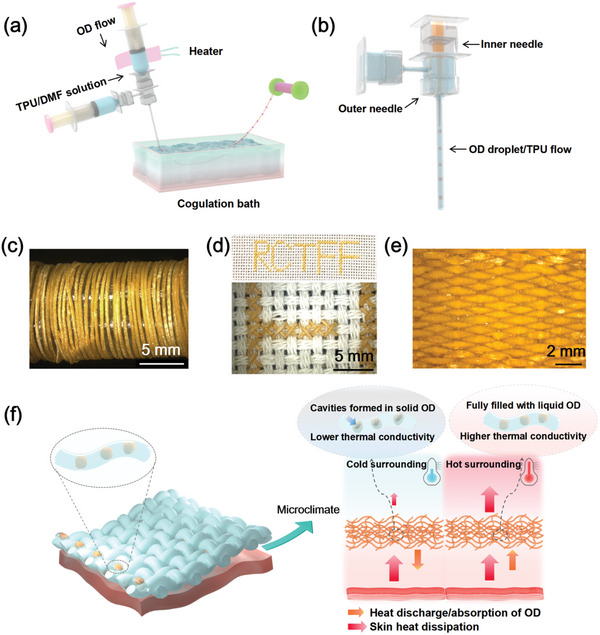
a) A schematic diagram of the droplet‐based microfluidic system for the fabrication of the TATFs, b) the coaxial needles for cellular microfluidic spinning, c) TATFs collected on a continuously automatic roller (scale bar = 5 mm), d) embroidered pattern of “RCTFF” (scale bar = 5 mm) using TATF, e) woven fabrics (scale bar = 2 mm) using the TATFs, f) temperature‐adaptive heat transfer through TATF fabric.

The novel cellular temperature‐adaptive thermoregulation filaments (TATFs) were fabricated using a droplet‐based microfluidic wet spinning system (**Figure** **1**a) with a coaxial needle having the inner needle's inner diameter of 150 µm and outer needle's inner diameter of 500 µm (Figure 1b), respectively. Melted octadecane (OD) liquid (dispersed phase) was injected through the inner needle and the dimethylformamide (DMF) solution of thermoplastic polyurethane (TPU) precursor (continuous phase) was injected through the outer coaxial needle. To ensure OD droplets were produced and distributed uniformly within the TPU/DMF flow, the flow rates of OD and TPU/DMF should be set within an appropriate range. To maintain OD in the liquid form before being injected into the coagulation bath, the syringes and the coaxial needles were heated to 40 °C using an electric resistance wire. In the coagulation bath containing deionized (DI) water, the TPU precursor was solidified by a simple and rapid solvent displacement between DMF and DI water, producing continuous filament which was then collected by a roller (Figure 1c). The filaments can be used for embroidering (Figure 1d) or weaving into fabrics (Figure 1e), demonstrating their feasibility for practical textile applications.

The mechanisms of temperature‐adaptive thermal regulation of TATF fabric are illustrated in Figure 1f. When the wearer moves from a hot to a cold environment with the temperature of OD in the fabric being decreased to below its phase transition temperature of 28 °C, liquid OD solidifies into solid forming vacuum cavities in the cellular OD cores and thereby reducing heat conduction apart from releasing a large amount of latent heat. On the contrary, when the wearer moves from the cold to the hot environment with the temperature of OD in the fabric being increased above its phase transition temperature of 28 °C, solid OD liquefies making the vacuum cavity in the cellular cores vanish, thereby increasing thermal conductivity apart from the short cooling effect due to latent heat absorption. Thus, the regulation of temperature‐adaptive thermal conductivity resulting from the dynamic volume changes of cavities in both cold and hot environments is long‐lasting without changes in the outer appearance of the filament, which further forms synergistic thermoregulation effect with transient release/absorption of latent heat when phase change material is used.

The droplet formation in co‐flow microfluidics^[^ ^22^ ^]^ is governed by inertial force, viscous force, and capillary force, which can be represented by the non‐dimensional numbers of Capillary number (*Ca* = *ηU*/*σ*, the ratio of viscous force to interfacial tension) of the continuous phase and the Weber number (*We* = *ρV* ^2^ *l*/*σ*, the ratio of inertial force to interfacial tension) of the dispersed phase.^[^ ^23^ ^]^ For calculating *We*, the characteristic length (*l*) is the inner diameter of the inner needle, *V* is the velocity of the liquid OD, *ρ is* the density of the liquid OD (*ρ* = 0.7770 g mL−1) and *σ is* the interfacial tension between liquid OD and TPU/DMF solution.^[^ ^18^ ^]^ For calculating the Capillary number (*Ca*), *η* is the viscosity of the TPU/DMF solution and *U* is the velocity of the continuous phase (TPU/DMF) solution. The interfacial tension (*σ*) between liquid OD and TPU/DMF solution was measured by Kruss, K100 to be 3.3910 mN m^−1^, and the dynamic viscosity (*η*) of the continuous phase (TPU/DMF solution) was measured by AMETEK Brookfield, DV2T to be 4200 cP.

**Figure 2:**
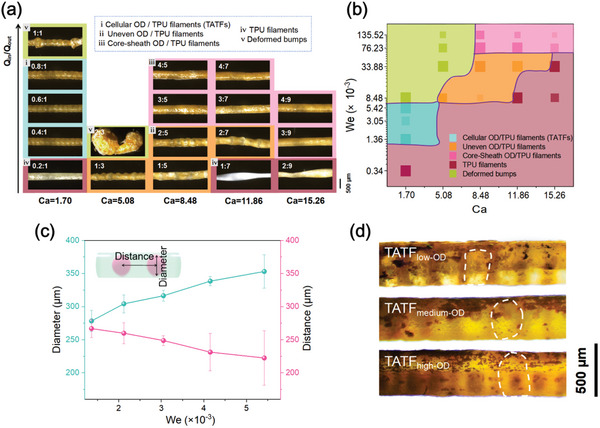
a) Photographs of OD/TPU filaments fabricated upon different flow ratios (scale bar = 500 µm). (i) The cellular OD/TPU filaments (TATFs) contained uniformly distributed OD particles; (ii) uneven OD/TPU filaments contained nonuniform OD particles; (iii) core–sheath OD/TPU filaments consist of continuous OD core and TPU sheath layer; (iv) TPU filaments are the ones in which no OD component can be encapsulated in the continuous phase of TPU solution; (v) deformed bumps mean OD and TPU components cannot form filament morphology. b) Phase diagram of OD/TPU filament morphologies with respect to *Ca* and *We*. c) Influence of *We* (*We* = 1.36, 2.12, 3.05, 4.15, 5.42×10^−3^) on the diameter of OD cells and the distance between adjacent capsules in the filament at *Ca* = 3.39. d) Photographs of TATFs with 32.4% (TATF_low‐OD_), 41.5% (TATF_medium‐OD_), and 52.3% (TATF_high‐OD_) OD loading ratios generated at *We* = 1.36, 3.05, 5.42 ×10^−3^ when *Ca* = 3.39. The OD core cells were marked by white boxes (scale bar = 500 µm).

To investigate the optimum condition for OD droplet generation in TATFs, different flow rates of the TPU solution and liquid OD were experimented. The flow rates of the continuous phase (viz. TPU solution) were set at 1, 3, 5, 7 and 9 mL h^−1^, corresponding to *Ca* = 1.70, 3.39, 5.08, 8.48, 11.86, 15.26, whereas the flow rate ratio between liquid OD and TPU solution (*Q*_in_ /*Q*_out_) varied from 0.2 to 1 (viz. corresponding to 0.34 × 10^−3^ < *We* < 135.52 × 10^−3^). **Figure** **2**a shows the photos of the filament morphology generated at different flow conditions. It can be seen that, at a low *Ca* number (*Ca* < 5.08), spherical OD capsules can be encapsulated in the TATFs uniformly at 0.4 ≤ *Q*_in_/*Q*_out_ < 1 (viz. 1.36 × 10^−3^ ≤ *We* < 8.48 × 10^−3^).

Figure 2b further illustrates the phase diagram of the TATF morphology with respect to *Ca* (continuous phase) and *We* (dispersed phase). It can be seen that stable cellular structure of TATFs can be manufactured at *Ca* < 5.08 and 1.36 × 10^−3^ ≤ *We* < 8.48 × 10^−3^; deformed bumps were generated at *Ca* < 8.48 and *We* ≥ 8.48 × 10^−3^; uneven OD/TPU filaments were produced at 5.08 ≤ *Ca* < 15.26 and 8.48 × 10^−3^ ≤ *We* < 76.23 × 10^−3^; core–sheath OD/TPU filaments were fabricated at *Ca* ≥ 8.48 and *We* ≥ 76.23 × 10^−3^; TPU filaments were formed in low and medium *We* regions depending on *Ca*, viz. *We* < 1.36 × 10^−3^ (*Ca* < 5.08), *We* < 8.48 × 10^−3^ (5.08 ≤ *Ca* < 11.86), *We* < 33.88 × 10^−3^ (11.86 ≤ *Ca* < 15.26), and *We* < 76.23 × 10^−3^ (*Ca* ≥ 15.26).

The diameters of cellular OD/TPU filaments (viz. TATFs) and OD cells in the TATFs as well as the distance between adjacent OD cells were characterized using Image J. It was found that, although the TATFs were fabricated at various *We* values, the filament diameter was almost constant at about 420.3 ± 3.7 µm regardless of *We* (Figure S1a,b, Supporting Information), which is smaller than the diameter of the polar bear fur‐like porous aerogel fiber in reference.^[^ ^24^ ^]^ Nonetheless, the OD cell diameter (278.4 ± 16.1 to 353.1 ± 25.2 µm) had a positive correlation with *We*, whereas the distance between adjacent OD cells (266.8 ± 13.6 to 222.4 ± 41.2 µm) had a negative correlation with *We* (Figure 2c). At *Ca* = 3.39, the TATFs produced at *We* = 1.36 × 10^−3^, 3.05 × 10^−3^, 5.42 × 10^−3^ had different OD loading rates of 32.4%, 41.5% and 52.3%, respectively (Figure 2d), which were denoted as TATF_low‐OD_, TATF_medium‐OD_, and TATF_high‐OD_. The dynamics of the melting process within the OD cells were recorded with video recording (Video S1, Supporting Information), where the volume of vacuum cavities in the cellular cells gradually decreased leading to increasing thermal conductivity. It should be noted that finer TATFs with diameters from 85 to 217 µm can also be fabricated by drawing the TATF with different roller speeds during filament collection. The cellular structure of these TATFs can be clearly seen in the optical microscope images (Figure S1c, Supporting Information).

### 2.2 Structural Analysis and Mechanical Properties of the TATFs

**Figure 3:**
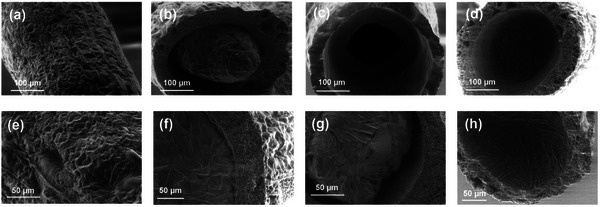
ESEM images of TATF_high‐OD_: a) filament surface, b) top, c) middle and d) bottom cross sections of cellular OD cells; higher magnification ESEM of e) outer surface, f) cross section, g) inter wall and h) inner cavity of the filament.

The environmental scanning electron microscope (ESEM) images of the outer surface of TATF_high‐OD_ (**Figure** **3**a,e) exhibited a smooth and dense TPU surface without apparent holes in the filament surface. Moreover, to verify the cellular structure of the TATFs, the filaments were first immersed in liquid nitrogen to preserve the filament structure and then cut off to produce the cross sections at the top, middle and bottom of an OD cell, which were then characterized by ESEM. The intact surface of solidified OD in the TPU filament (Figure 3b) and the smooth inner surface of the cell (Figure 3d) suggested an effective discrete encapsulation of OD and the formation of impeccable cellular structure. Furthermore, a cavity was observed in the middle of the solid OD cell (Figure 3c), which was generated by the volume shrinkage of OD when transformed from liquid to solid. The dense TPU layer can be observed by the ESEM of the outer surface (Figure 3e), cross section (Figure 3f), inter wall (Figure 3g) and cavity bottom (Figure 3h) of the filament.

In order to examine whether there can be any leakage of liquid OD from the cellular TATFs, we conducted the following experiment. The cellular TATFs and the conventional OD/TPU core–sheath filaments were pasted on a piece of filter paper with a pattern of “SFT” at the ambient temperature of 20 °C, when the OD was in solid form. Thereafter, the assembly was heated to 40 °C to melt OD. It was observed (Figure S2, Supporting Information) that, a large amount of OD leaked out from the conventional core–sheath filament onto the filter paper, whereas no noticeable OD leakage from the cellular TATFs was discovered, indicating excellent encapsulation of OD core in the cellular TATFs. The issue of OD leakage was further evaluated during the stretching process by rubbing the TATF_high‐OD_ vigorously using filter paper when the TATF was heated by red light irradiation. And, no melted OD leakage was detected on the filter paper (Figure S3c,d and Video S2, Supporting Information), indicating that the TPU polymer network remodeled by microfluidic spinning can tackle the leakage problem even TPU chains shifted under tension, which provided a promising OD encapsulation strategy to generate temperature‐adaptive vacuum cavities for wearing in motion. Additionally, the fabrics made of TATFs were washed using detergent at 60 °C for 3 times (Figure S6c, Supporting Information), which showed almost no mass loss illustrating that no leakage of OD even under normal washing. Furthermore, there was no visible change in the morphology of TATFs while OD melted in these experiments. This is because the segments of TPU between adjacent OD cells can provide strong support when OD melts. In addition, the size of the liquid OD cell is on a micrometer scale, making it difficult for the OD cell to deform.

**Figure 4:**
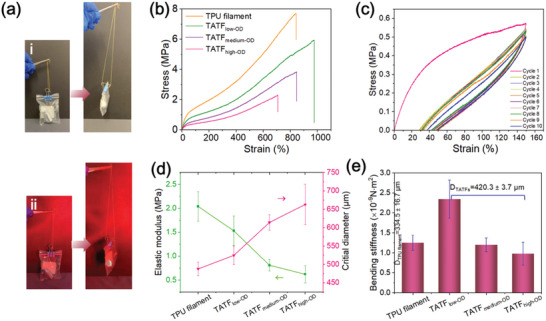
a) Photographs of the high stretchability of TATF with high OD content in (i) solid OD state at about 22 °C and (ii) melting OD state at about 30 °C under a weight loading of 50 g. b) Stress–strain curves of TATFs in solid OD state. c) Cyclic stress‐strain curves of TATF_high‐OD_ at a fixed strain of 150% for 10 cycles in a solid OD state. d) Elastic modulus and critical diameter of filaments with different OD content. e) Bending stiffness of filaments with different OD content.

The mechanical strength and extensibility of TATF was demonstrated by loading a weight of 50 g under solid OD state (viz. approximately at 22 °C) and liquid OD state (viz. approximately at 30 °C) (**Figure** **4**a), demonstrating the robustness of the TATF. The mechanical properties of the TATFs fabricated with various OD loading ratios (viz. TATF_low‐OD_, TATF_medium‐OD,_ and TATF_high‐OD_) were further tested by a tensile tester (Instron, 5566 UTM) at 22 °C (viz. solid OD state) and 30 °C (viz. melting OD state). The results are shown in Figure 4b and Figure S3b (Supporting Information). The cellular structure can be clearly observed during stretching (Figure S3a, Supporting Information). As the OD loading ratio increased from 32.4% (TATF_low‐OD_) to 52.3% (TATF_high‐OD_), the TATFs exhibited decreased tensile strength from 6.4 ± 0.7 to 2.1 ± 0.4 MPa (similar to that of the polar bear fur‐like porous aerogel fiber),^[^ ^24^ ^]^ but slightly increased tensile strain from 640% to 960% at break. In contrast, the pure TPU filament had a slightly higher tensile strength σ_t_ = 7.1 ± 0.7 MPa with the tensile strain at break *ε*_t_ = 810%, which is induced by the stronger block copolymerization network of the hard and soft segments in TPU. The hard segments supplied TPU chains with rigidity, while the soft segments contributed flexibility to TPU chains.^[^ ^25^ ^]^ The blocking of OD cells between TPU polymer networks directly reduced the connectivity of the hard segments, as reflected by the decline in tensile strength with the increase in OD content. On the other hand, the filament tensile strain increased first and then decreased with the increasing OD content. Moreover, the durability of TATFs was demonstrated by a cyclic tensile test upon loading and unloading with a strain setting of 150% for 10 times (Figure 4c). Except for the first deformation, tensile curves almost maintained the same route during the remaining cycles. This is because the physical structure becomes stable after the destruction of irreversible composition in polymer chains in the first tensile loading–unloading loop.^[^ ^26^ ^]^

The stress–strain curve of TATFs with melting OD displayed that the yield limit negatively correlated with OD content (Figure S3b, Supporting Information), because of the different degrees of permanent damage at weaker points in the TPU network upon stretching. At 32.4% OD content, TATF_low‐OD_ exhibited almost a linear stress–strain curve accompanied by lower Young's modulus (viz. the initial slope value of the linear region in the stress–strain curve). At higher (41.5% and 52.3%) OD contents, TATF_medium‐OD_ and TATF_high‐OD_ behaved more rigidity (viz. higher Young's modulus) at the initial extension, then softened with continued stretching and finally increased their rigidity again before fracture. This can be attributed to the uneven stress distribution in TATFs formed by the melted OD droplets.

In order to avoid next‐to‐the‐skin prickling discomfort for apparel applications, filaments should be finer than the critical diameter, which is related to the filament Young's modulus (Equation S1, Supporting Information). The calculated critical diameters of TATF_low‐OD_, TATF_medium‐OD,_ and TATF_high‐OD_ are 525, 613, and 663 µm, respectively (Figure 4d). Therefore, the diameters (420 µm) of TATF_low‐OD_, TATF_medium‐OD,_ and TATF_high‐OD_ are finer than their respective critical diameter, indicating that they will not cause prickling discomfort for next‐to‐the‐skin apparel.^[^ ^27^ ^]^

The bending stiffness (*R*_f_) of the TATFs displayed a negative dependence on the OD loading ratio. This is understandable as *R*_f_ is proportional to Young's modulus (*E*) and filament diameter (*D*) to the power of 4 (Figure 4e, Equation S2, Supporting Information). As the TATF diameters are almost constant regardless of the OD loading rate, the effect of OD loading rate on bending stiffness is therefore similar to that on Young's modulus. The slightly lower bending stiffness of pure TPU filament (1.3 ± 0.2 × 10^−9^ N m^2^) shown in Figure 4d was primarily attributed to its finer diameter (viz. 334.5 ± 16.7 µm).

### 2.3 Thermal Properties of the TATFs

**Figure 5:**
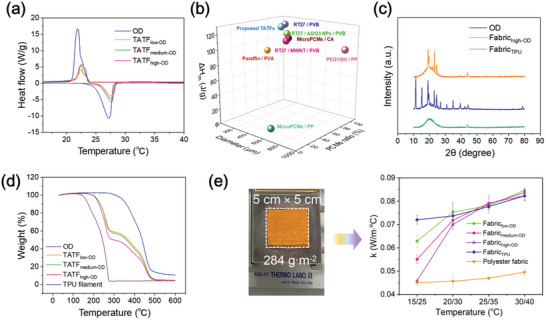
a) DSC curves of pure OD and TATFs. b) Comparison of the melting enthalpy of the proposed TATF_high‐OD_ with that of previously reported core–sheath PCM filaments in refs. [28, 30, 31, 32, 33, 34, 35] c) XRD spectra of OD, TPU filament‐woven fabric (Fabric_TPU_) and TATF_high‐OD_‐woven fabric (Fabric_high‐OD_). d) TGA curves for the decomposition of OD, TPU filament and TATF_high‐OD_. e) The testing photo of a sample with a surface density of 284 g m^−2^ and the thermal conductivities of polyester fabric, Fabric_TPU,_ and the fabrics woven by TATF_low‐OD_ (Fabric_low‐OD_), TATF_medium‐OD_ (Fabric_medium‐OD_) and TATF_high‐OD_ (Fabric_high‐OD_), respectively.

The cellular OD ratio of TATFs can be evaluated by Differential Scanning Calorimetry (DSC) curves.^[^ ^28^ ^]^ The starting/ending phase change temperature, as well as the phase change enthalpy, for OD melt/solidification was recorded to characterize the thermal properties of TATFs with different OD content. The TATFs with different OD content exhibited almost the same melting and solidification temperatures with the melting temperature ranging from 25.5 ± 0.2 to 28.4 ± 0.1 °C and the solidification temperature ranging from 23.9 ± 0.1 to 21.5 ± 0.1 °C. Compared with pure OD (melting temperature: 24.6 to 28.0 °C and solidification temperature: 23.6 to 20.9 °C), TATFs presented similar behavior upon temperatures of melt and solidification (**Figure** **5**a, Table S1, Supporting Information). This indicated that the OD encapsulated in the TATFs during the microfluidic spinning had a high purity which is crucial for generating vacuum cavities in OD cells. In addition, the cellular structure does not affect the phase transition of the encapsulated OD evidenced by homologous phase change behaviors in all groups of TATFs, which is also consistent with that of pure OD. Moreover, no phase change lag effect of the cellular OD was discovered in TATFs, which shows the cellular OD capsules have no undesirable supercooling phenomenon.^[^ ^29^ ^]^ Furthermore, the melting enthalpy (Δ*H*_m_) of TATFs, a prominent indicator for their thermal performance, was calculated through DSC measurement. They were 79.0, 101.3 ± 3.1, and 128.5 ± 9.7 J g^−1^ for TATF_low‐OD_, TATF_medium‐OD_ and TATF_high‐OD_, respectively, which was proportional to the content of OD. If the OD mass fraction in the filament was 100%, the melting enthalpy of the filament would be that of pure OD (243.8 J g^−1^). By comparing the melting enthalpy, the encapsulated OD contents within TATF_low‐OD_, TATF_medium‐OD_ and TATF_high‐OD_ were 32.4 ± 1.4%, 41.5 ± 1.3% and 52.3 ± 3.9%, respectively. Moreover, the good thermal stability of TATF_high‐OD_ was illustrated by loop DSC curves for 10 cycles of heating and cooling, which showed extremely similar routes in each cycle (Figure S4a, Supporting Information). We also did the DSC test for TATF_high‐OD_ after washing using detergent at 60 °C (Figure S4b, Supporting Information), which showed Δ*H*_m_ = 103.52 and 101.40 J g^−1^ (viz. the residual mass ratio was 98%) before and after washing, respectively, suggesting that OD can be well embedded in the cellular and the loss of encapsulated OD was negligible. Thus, the fabrics woven by TATFs are capable of repeatable and durable thermoregulation. Compared with previously reported core–sheath filaments (e.g., PCM composite filaments), the TATFs exhibited a larger OD loading ratio (52.3%) with excellent melting enthalpy (128.5 J g^−1^), which is profitable to result in larger vacuum cavities in OD cells for wearing thermal comfort (Figure 5b).^[^ ^28^, ^30^, ^31^, ^32^, ^33^, ^34^, ^35^ ^]^

The pure TPU filaments and TATFs were further woven into fabrics in the size of 5 cm × 5 cm, and denoted as Fabric_TPU_, Fabric_low‐OD_, Fabric_medium‐OD_ and Fabric_high‐OD_, for fabrics made of TPU filament, TATF_low‐OD_, TATF_medium‐OD_ and TATF_high‐OD,_ respectively. Similar sharp diffraction peaks between Fabric_high‐OD_ and pure OD were shown at 2*θ* = 19.2, 19.6, 23.3, and 24.6 in the X‐ray diffraction (XRD) spectra (Figure 5c), illustrating that the Fabric_high‐OD_ had excellent crystallization properties. The thermal stability of the pure OD, TPU filament and TATFs were further investigated by Thermogravimetric analysis (TGA) (Figure 5d). The TGA curves of TATFs can be divided into two stages between the curves of OD and TPU. The first stage witnessed a rapid descent from 195 to 282 °C, which was caused by drastic decomposition of OD from 141 to 276 °C. A second stage tardier decline from 282 to 510 °C can be observed, which was attributed to the decomposition of the hard and soft segments of TPU with different descent rates. It is noteworthy that little or no weight loss occurred before 190 °C in all groups of TATFs, indicating high thermal stability and extensive applicability for wearing applications. Moreover, the degradation rate of TATFs depended on the OD content, with TATFs of higher OD content having a more rapid decomposition rate.

The thermal conductivities (*k*) of the TATF fabrics (viz. Fabric_low‐OD_, Fabric_medium‐OD_, and Fabric_high‐OD_), a pure TPU fabric (Fabric_TPU_) and a conventional polyester fabric were measured using the modified KES (Kawabata Evaluation System) Thermo Labo‐II (Figure S5, Supporting Information) under the cold plate temperature (*T*_c_)/hot plate temperature (*T*_h_) of 15/25 °C, 20/30 °C, 25/35 °C, and 30/40 °C, respectively. At the low temperature (*T*_c_/*T*_h_ = 15/25 °C), TATF fabrics exhibited significantly lower thermal conductivity than that of Fabric_TPU_, due to the vacuum cavities created as a result of the shrinkage of OD in the solidification process (Figure 5e). When the temperature increased from 20 °C (viz. *T*_c_/*T*_h_ = 15/25 °C) to 35 °C (Viz. *T*_c_/*T*_h_ = 30/40 °C), the thermal conductivities of TATF fabrics rose sharply first, then more gradually. Comparing the TATF fabrics with varying OD contents, increasing the OD content has a positive effect on the temperature‐adaptive thermal conductivity, with an increase of 35% for Fabric_low‐OD_, 50% for Fabric_medium‐OD_, and 83% for Fabric_high‐OD_. In contrast, with the same temperature increase, the thermal conductivity of the pure TPU fabric (viz. Fabric_TPU_) only increased by just 12% and a conventional polyester fabric increased by just 10%. The significantly higher increase in the thermal conductivity of the TATF fabric as the temperature increases is attributed to the filling of vacuum cavities by increasing volume of Octadecane (OD) during melting (Video S1, Supporting information), but not by the increased thermal conductivity of Octadecane (OD), as the thermal conductivity of OD in the liquid state at a temperature above 28 °C is lower than that in the solid state at a lower temperature.^[^ ^36^ ^]^

### 2.4 Temperature Responsiveness of the TATF‐Woven Fabrics

To evaluate the temperature‐adaptive thermal performance of TATF‐woven fabrics, an infrared camera was used to record the temperature variation of TPU fabrics and TATF‐woven fabrics with different OD loading ratios in both cooling and heating scenarios (Figure S7, Supporting Information). The fabric with an initial high or low temperature was put onto a cold or hot copper plate with a constant temperature controlled by a water bath, which simulated different thermal environments upon the fabrics. Then, the infrared thermal images of the fabrics were captured within 7 min.

**Figure 6:**
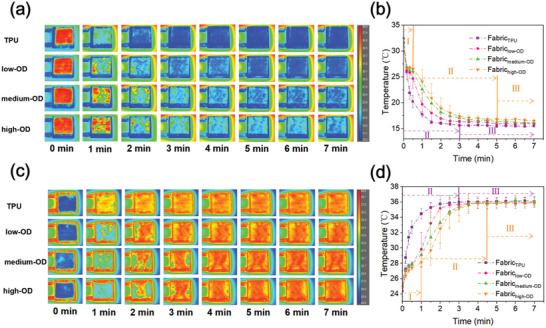
a) Time‐sequential infrared images and b) transient average temperature of different fabrics in the cooling process, and c) time‐sequential infrared images and d) transient average temperature of different fabrics in the heating process.

In the cooling process, all fabrics were first heated to 40 °C before putting on the cold copper plate with a constant temperature of 15 °C (**Figure** **6**a). After being placed on the copper plate, the temperature of Fabric_TPU_ plunged rapidly and then cooled down to the lowest temperature (viz. when the color of the sample no longer changes) in the shortest time (viz. 2.5 min) among all groups of fabric. For Fabrics woven by TATFs, the rate of temperature decline decreased with the increasing OD content, as evidenced by the thermal images in Figure 6a. Furthermore, the temperature variation of TATF‐woven fabrics can be divided into three stages (Figure 6b). In stage I, the temperatures first dropped fast due to the sensible heat release of the fabric and then decreased slowly until reached a transient plateau caused by the starting of the latent heat release of the cellular OD. Then, the temperature decreases to steady situations denoted in stage II, in which the OD completely solidifies gradually. Finally, the fabric maintained the final temperature for the rest of the time (stage III). In comparison, Fabric_TPU_ just had two temperature drop stages, viz. the rapid temperature decline (stage II) without OD solidifying and temperature stabilization (stage III), presenting a larger temperature descending rate and a lower period (about 2.5 min) to reach the stable temperature. In contrast, the TATF‐woven fabrics had lower cooling rates with longer cooling times of 3, 4, and 4.5 min for Fabric_low‐OD_, Fabric_medium‐OD_, and Fabric_high‐OD_, respectively, to reach the steady conditions. Furthermore, the TATF‐woven fabrics also had higher steady temperatures (Fabric_low‐OD_: 15.93 ± 0.09 °C, Fabric_medium‐OD_: 16.28 ± 0.12 °C, and Fabric_high‐OD_: 16.67 ± 0.14 °C) than that of Fabric_TPU_ (15.48 ± 0.05 °C). Meanwhile, the higher the OD loading ratio, the higher the stable temperature. This was because fabric thermal conductivity decreases with the increasing OD loading ratio in cold environments (Figure 5e). Consequently, Fabric_high‐OD_ can significantly reduce heat dissipation in cold environments due to the vacuum cavities formed within the cellular solid OD.

In the heating process, the fabrics to be tested were stored at room temperature at about 21 °C, which were then rapidly placed on the copper plate at a temperature of 37 °C (Figure 6c). Figure 6c further shows that the Fabric_TPU_ had a larger temperature increasing rate than that of TATF‐woven fabrics. In Figure 6d, the temperature of the fabrics woven by TATFs raised rapidly until a short plateau was caused by latent heat absorption of the cellular OD (stage I). When OD completely melts, temperature sharply grows until the proximity of stable temperature (stage II). Finally, the fabric temperature reached the steady state and maintained the plateaus (stage III). In comparison, the Fabric_TPU_ possessed two stages (viz. stage II and stage III) because of the deficiency of OD latent heat regulation. As a result, the Fabric_TPU_ showed the largest rate of temperature rise with the shortest time (3 min) to achieve a stable temperature. In contrast, TATF‐woven fabrics had a lower temperature increasing rate and longer time to reach the steady state (viz. 3.5 min for Fabric_low‐OD_, 4 min for Fabric_medium‐OD_ and 5 min for Fabric_high‐OD_).

In brief, the fabric made by TATFs can exhibit temperature adaptive thermal conductivity in response to the environmental temperature, which can preserve body heat in cold environments and facilitate body heat dissipation in hot environments due to the thermoresponsive vacuum cavities in the cellular cores of the TATFs. Therefore, the dynamic cavities in OD cells can persistently function in regulating body heat dissipation without changing the appearance of the fabric, which can provide an efficient and lasting passive body thermal management without affecting the wearing comfort.

## 3 Conclusions

Functional fibers/filaments with prolonged passive temperature‐adaptive thermoregulation are highly desirable for personal thermal management. In this study, a co‐flow droplet‐based microfluidic strategy was used to produce the TATFs with cellular OD capsules in TPU because the temperature‐adaptive OD volume change can form thermoresponsive vacuum cavities in the cellular OD, resulting in temperature‐adaptive thermal conductivity of the filament. The proposed TATFs can encapsulate a large amount of high‐purity OD for the creation of vacuum cavities and demonstrate robust strength, stretchability, and extensibility for wearable applications. Apart from the thermoregulation by the absorption/release of OD's latent heat, the TATFs had a lower thermal conductivity to preserve heat in cold environments, due to the formation of vacuum cavities inside the solid OD cells, and a higher thermal conductivity in hot environments to facilitate heat dissipation, because of the disappearance of the vacuum cavities. As a result, the fabrics woven by TATFs displayed temperature‐adaptive thermoregulation response to the environmental temperature for both cooling and heating scenarios, which can provide thermal comfort to the wearer and alleviate heat/cold stress in various environments. This work contributes to the development of advanced thermal management garments and the promotion of a low‐carbon society.

## Conflict of Interest

The authors declare no conflict of interest.

## Supporting information



Supporting Information

Supplemental Video 1

Supplemental Video 2

## Data Availability

The data that support the findings of this study are available from the corresponding author upon reasonable request.
