# Supplementary material for: Cellular Core/Sheath Filaments with Thermoresponsive Vacuum Cavities for Prolonged Passive Temperature‐Adaptive Thermoregulation
Source: Adv Sci (Weinh). 2025 Jan 7;12(8):2412448. doi: 10.1002/advs.202412448 (PMC11848530; doi:10.1002/advs.202412448)
Supplement: Supplementary file 1 — Supporting Information [file ADVS-12-2412448-s001.docx]

**Supporting Information**

**Cellular Core/Sheath Filaments with Thermoresponsive Vacuum Cavities for Prolonged Passive Temperature-adaptive Thermoregulation**

Jiayi Sui^a, b, #^, Shoukun Jiang^a, b, #^, Jinhao Peng^e^, Zhanxiao Kang^a, b, c,^ **^*^**, Jintu Fan^a, b, d,^ **^*^**

^a^School of Fashion and Textiles, The Hong Kong Polytechnic University, Hung Hom, Kowloon, Hong Kong, China

^b^Research Centre of Textiles for Future Fashion, The Hong Kong Polytechnic University, Hung Hom, Kowloon, Hong Kong, China

^c^Future Intelligent Wear Centre, The Hong Kong Polytechnic University, Hung Hom, Kowloon, Hong Kong, China

^d^Research Institute of Sports Science and Technology, The Hong Kong Polytechnic University, Hung Hom, Kowloon, Hong Kong, China

^e^College of Chemistry and Environmental Engineering, Shenzhen University, Shenzhen 518055, PR China

^#^These authors (J. Sui and S. Jiang) contributed equally.

^*^Corresponding authors: [z.x.kang@polyu.edu.hk](mailto:z.x.kang@polyu.edu.hk) (Z. Kang); [jin-tu.fan@polyu.edu.hk](mailto:jin-tu.fan@polyu.edu.hk) (J. Fan)

**Calculation of bending stiffness and critical diameter:**

According to equation S1, the critical diameter (*D*_critical_) of the filament can be calculated by:

$D_{\mathrm{critical}}=4\sqrt[4]{\frac{l^{2}P_{E}}{\pi^{3}E}}$ (Eq. S1)

where *E* is Young’s modulus of filament, *l* (2 mm) and *P*_E_ (0.75 mN) is the length and critical buckling load of filament [27].

In accordance with the equation S2, bending stiffness (*R*_f_) can be calculated by Young’s modulus (*E*) and diameter (*D*) of filament:

${R_{f}=\pi ED^{4}}/{64}$ (Eq. S2)

**Calculation of thermal conductivity:**

Thermal conductivity (*k*) can be obtained by equation S3:

$k=\frac{W\cdot d}{A\cdot\Delta T}$ (Eq. S3)

where *W* is related to the amount of heat transferred from the heat plate through the sample to the cold plate, which can be obtained from instrument readings, *d* is the thickness of the sample, *A* is the area of the heat plate (viz. the area of 5 × 5 cm) and ∆*T* is the temperature difference between heat plate and cold plate (viz. ∆*T =* 10 ℃ in this study).

Table S1. Phase change behavior of the samples.

| Samples | Melting Process | | | Crystallization process | | |
| --- | --- | --- | --- | --- | --- | --- |
|  | *T*_mo_  [°C] ^a)^ | *T*_me_  [°C] ^b)^ | Δ*H*_m_  [J g^-1^] ^c)^ | *T*_co_  [°C] ^d)^ | *T*_ce_  [°C] ^e)^ | Δ*H*_c_  [J g^-1^] ^f)^ |
| OD | 24.6 | 28.0 | 243.8 | 23.6 | 20.9 | 256.06 |
| TATF_low-OD_ | 25.7 | 28.3 | 79.0 | 24.0 | 21.5 | 96.88 |
| TATF_medium-OD_ | 25.6 | 28.5 | 101.3 | 23.9 | 21.4 | 111.74 |
| TATF_high-OD_ | 25.3 | 28.3 | 128.5 | 23.8 | 21.5 | 144.81 |

^a)^ Onset melting temperature; ^b)^end melting temperature; ^c)^melting enthalpy; ^d)^onset crystallization temperature; ^e)^crystallization peak; ^f)^end crystallization temperature; ^g)^crystallization enthalpy.

**Materials and methods**

*Materials:*

Thermoplastic polyurethane (TPU) Elastollan^®^ was purchased from BASF. Octadecane (OD) and Span^TM^ 80 were purchased from Aladdin. N, N-Dimethylformamide (DMF) and ethanol were obtained from AQA. Sudan Ⅰ was purchased from Macklin. All chemicals are at least analytical reagent grade.

*Fabrication of coaxial needle:*

The coaxial needle was composed of stainless steel needles of 17 G (viz. inner diameter: 1.05 mm, outer diameter: 1.5 mm), 21 G (viz. inner diameter: 0.50 mm, outer diameter: 0.8 mm) and 30 G (viz. inner diameter: 0.15 mm, outer diameter: 0.3 mm) with the lengths of 6.5 mm, 51 mm, and 6.5 mm, respectively. The needle of 17 G, the inlet of the outer channel, was first welded to the side face of the 21 G needle which was used as the outer channel, and then the 30 G needle, the inner channel, was embedded in the 21 G needle after accurate calibration, which is important to structural stability of cellular OD/TPU filaments.

*Preparation of precursor solutions for spinning:*

Firstly, 8 g TPU powder was dispersed in 20 mL DMF through magnetic stirring under continuous heating of 100 ℃ for 2 h. Then, 5% (W/V) of Span 80 was added to the TPU/DMF solution and stirred until dissolution to prepare a homogenous precursor solution. OD was heated to a liquid state at a temperature of 40 ℃ and then dyed by adding appropriate Sudan Ⅰ for visual observation in microfluidic spinning.

*Fabrication of TATFs using droplet-based microfluidic spinning:*

The TATFs were fabricated through microfluidic spinning using a coaxial needle, and there is a sufficient difference in the lengths of the inner and outer channels to ensure that stable OD droplets were encapsulated in TPU/DMF flow. The syringes loaded with TPU/DMF solution and liquid OD were fixed on the injection pump, respectively, where both syringes were connected to the coaxial needle through the Teflon tube. Moreover, the entire microfluidic platform was heated to 40 ℃ by a temperature control system edited by Arduino. For TATFs that we used in further experiments, the TPU/DMF solution was injected into the outer channel with a constant extrusion rate of 2 mL∙h^-1^, and the dyed OD solution was injected into the inner channel with extrusion rates of 0.4, 0.6, 0.8 mL∙h^-1^, respectively. Simultaneously, the coaxial needle was injected into a coagulation bath filled with pure water, in which the TATFs can be solidified by solvent exchange between water and DMF, followed by a soak of 5 minutes for adequate solidification of filaments. Finally, the TATF was collected and dried on a roller.

*Preparation of TATFs-woven fabrics:*

The testing fabrics were woven by TPU filament, TATF_low-OD_, TATF_medium-OD_, TATF_high-_OD, respectively as weft and cotton filament with a diameter of 0.18 mm as warp. The fabrics were woven with appropriate linear density using a manual weaving machine.

*Modification of KES Thermo labo II:*

The unmodified KES Thermo labo II was composed of a heat plate as the upper cover and a cold plate as a subplate, in which heat was transmitted from the hot plate through the sample to the cold plate with a temperature difference of 10 ℃. The temperatures of both hot and cold plates were controlled by constant temperature systems. The accuracy of the original Thermal Labo-II cannot meet the requirements to evaluate the thermal conductivity at various testing temperatures from 15 / 25 ℃ to 30 / 40 ℃, due to the heat dissipation from the hot plate to the ambient environment. The lost heat cannot be calculated by the Thermal Labo-II system, thereby leading to errors in thermal conductivity measurement, especially at higher temperatures with larger temperature differences between the hot plate and ambient environment. Therefore, the KES-Thermo Labo-II was modified to prevent heat loss at high temperatures. The instrument was modified by introducing a heating slice around the hot plate with negligible thickness, in which the heating slice was controlled by a microcontroller unit (MCU) using Arduino where PID algorithm controls the on/off of the DC power supply with a constant voltage of 20 V. The temperature of the heating slice can be accurately set to be the same as that of the hot plate when testing the samples at various cold plate temperatures (*T*_c_) and hot plate temperatures (*T*_h_) of 15 / 25 ℃, 20 / 30 ℃, 25 / 35 ℃, 30 / 40 ℃.

**Characterization**

*Measurement of dynamic viscosity:*

The dynamic viscosity measurement was carried out for the TPU precursor solution under a viscometer (AMETEK Brookfield, DV2T) at 40 ℃.

*Measurement of interfacial tension between TPU solution and liquid OD:*

The interfacial tension was tested by a force tension meter (Kruss, K100).

*Environmental Transmission Electron Microscope (ETEM):*

The morphology and microstructure were obtained by scanning electron microscopy (Thermo Scientific, Quattro S) to characterize the cross section of TATFs and explore the cavity structure caused by the solidification of OD and the internal geometric distribution of the OD in TATFs.

## *Differential Scanning Calorimetry (DSC):*

## DSC was conducted on a Differential Scanning Calorimeter (Mettler Toledo, DSC3) with a heating and cooling rate of 5 ℃∙min^-1^ from 10 to 50 ℃ under nitrogen atmosphere, which was used to determine the melting and crystallization temperatures as well as latent heat of OD and TATFs in the heating and cooling cycles.

*Thermal Gravimetric Analyzer (TGA):*

Thermal stability was performed by Thermogravimetric Analyzer (Mettler Toledo, TGA/DSC 3+) with a heating rate of 20 ℃·min^-1^ from 30 to 600 ℃, which was used to determine the degradation temperatures in various degradation stages of OD, TPU filament and TATFs.

*Measurement of thermal conductivity:*

KES-Thermo Labo II (Kato Tech, KES-F7) was used to measure the thermal conductivity of fabrics woven by TPU filaments and TATFs as well as common polyester fabric under various environmental temperatures.

*Tensile test of filaments:*

A Tensile Tester (Instron, 5566 UTM) was used to measure the tensile strength and Young’s modulus of the TPU filament and TATFs at a solid/melting OD state. The common stress-strain curves were conducted with the tensile rate of 10 mm·min^-1^ and filament length of 2 cm. The cyclic tensile curves were tested with the tensile rate of 50 mm·min^-1^ and the constant strain of 150%.

*Infrared camera:*

The infrared images were captured by an infrared camera (FLIR, A600-series) in the environment bin with a temperature of 21 ℃ and humidness of 70% RH. The TATF-woven fabrics were put on a copper plate which was connected to a water bath with a constant temperature that simulated the external environment of fabrics. The infrared images of Fabric_TPU_ and TATF-woven fabrics at different time points were processed by the data acquisition system FLIR ResearchIR Max 4, where the area of fabric was selected by a frame to evaluate the average temperature of the fabric.

**
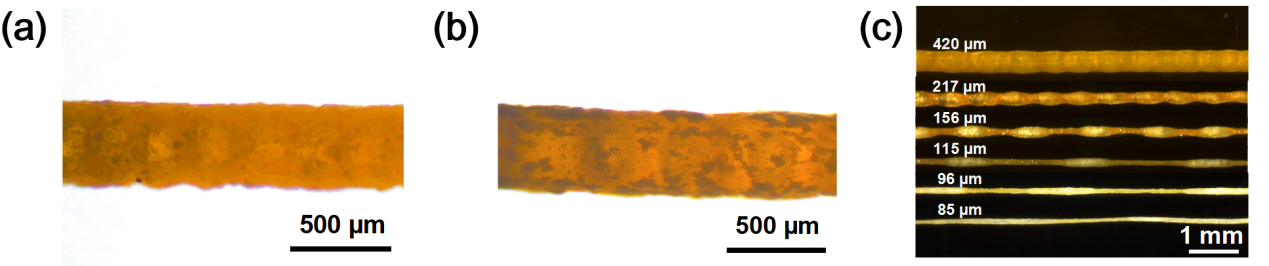
**

Fig. S1. Optical microscope photographs of TATFs with a constant *Ca* = 3.39 (corresponding to TPU phase velocity of 2 mL∙h^-1^) at different *We* of (a) 2.12 × 10^-3^ (corresponding to OD phase velocity of 0.5 mL∙h^-1^), and (b) 4.15 × 10^-3^ (corresponding to OD phase velocity of 0.7 mL∙h^-1^). (c) Optical microscope photographs of finer TATFs by drawing the thick filament.


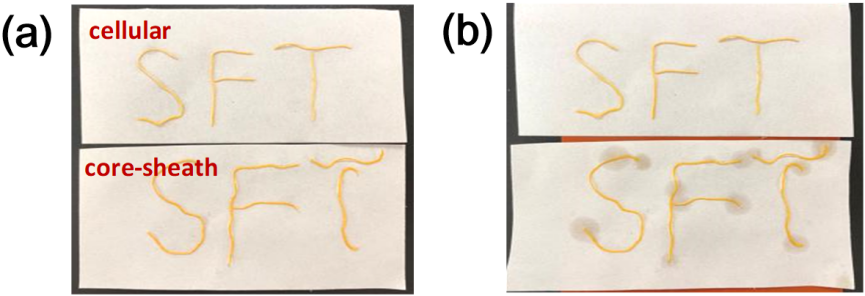


Fig. S2. Photographs of fractured TATFs and conventional core-sheath filaments in (a) solid OD state and (b) liquid OD state.


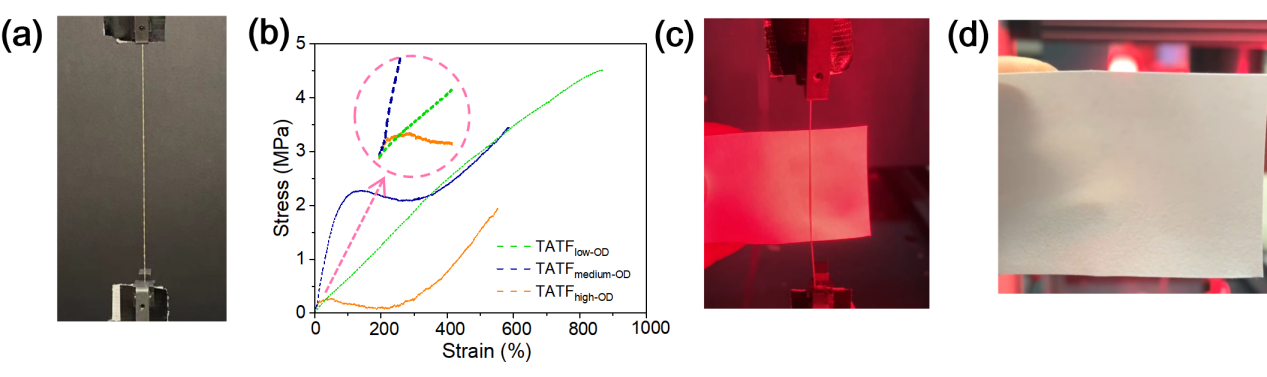


Fig. S3. Photograph of (a) TATF_high-OD_ during stretching with the solid state of OD. (b) Stress-strain curves of TATFs in liquid OD state. (c) TATF_high-OD_ with the liquid state of OD under red-light heating during stretching while rubbing with a filter paper. (d) Filter paper after rubbing with TATF_high-OD_.


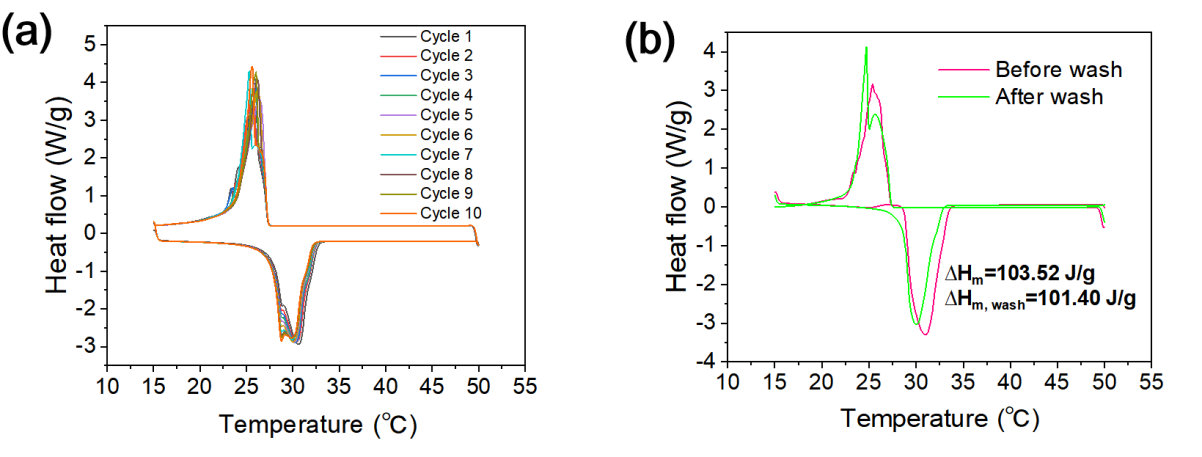


Fig. S4. DSC curves of TATF_high-OD_ before and after washing by detergent.


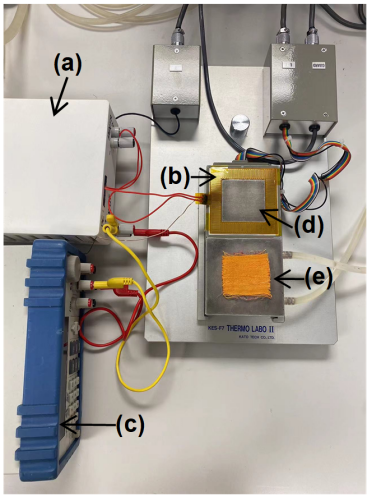


Fig. S5. Photograph of the modified KEF-Thermal Labo II for thermal conductivity measurement: (a) microcontroller unit (MCU) using Arduino, (b) heating slice, (c) DC power, (d) hot plate, and (e) cold plate.

**
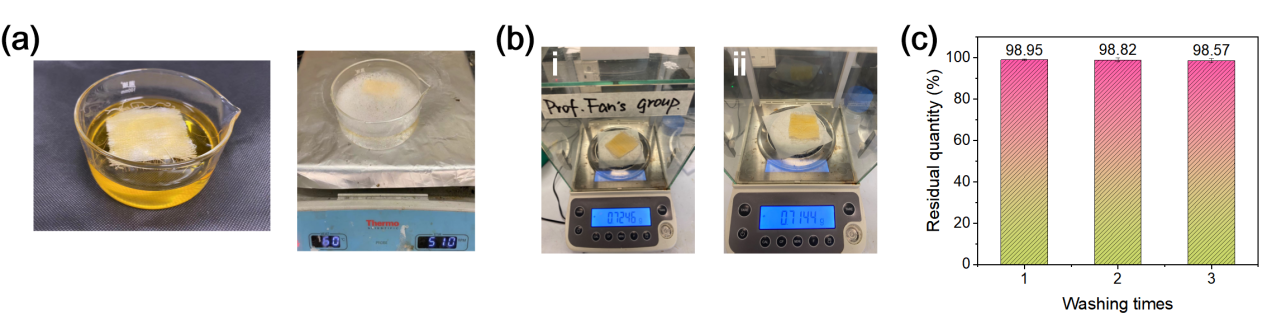
**

Fig. S6. Photographs of (a) Fabric_high-OD_ washed in ethanol (viz. dissolve OD on the surface using detergent at 60 ℃). (b) The fabric mass (ⅰ) before (viz. 0.7346 g) and (ⅱ) after (viz. 0.7144 g) washing. (c) Fabric_high-OD_ washed in detergent for 3 times at 60 ℃. There is almost no loss in fabric weight.


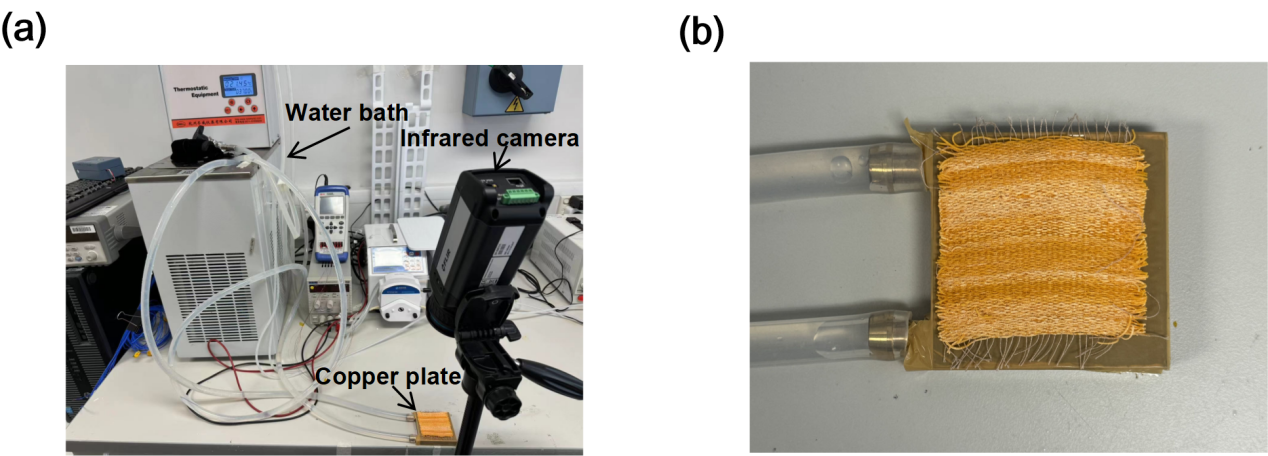


Fig. S7. Photographs of the (a) device for infrared thermal images, and (b) the copper plate with a fabric sample placed on it.
